# Landolfi’s Treatment of Cancer

**Published:** 1857-01

**Authors:** 


*Landolfi's Treatment of Cancer*.—M. Landolfi's mode of treating cancer having gained considerable notoriety in Austria, he repaired some time since to Paris, in order to induce the Surgeons of that capital to endorse the favorable opinions expressed by some of the Vienna practitioners. The French Hospital Surgeons accordingly appointed a committee of their body to examine into the ability of the claim, and this was done by assigning M. Landolfi a certain number of patients at the Salpêtrière. The committee, after watching the results of his treatment of these cases, has just made its report, and the following are the conclusions arrived at. From these it would seem that the remedy is destined to fall into the oblivion that has entangled so many of its predecessors.

1. M. Landolfi's method is made up of both local and internal treatment.

2. The latter, which consists in the administration of chloride of bromine, does not possess the slightest special therapeutical value in the treatment of cancer.

3. The local treatment consists in the application of the following caustic: Chloride of bromine, 3 parts; chloride of zinc, 2 parts; chloride of antimony, 1 part; liquorice powder, 1 part.

4. Of these substances, the chloride of zinc and chloride of antimony, have been long known and employed as caustics. These two chlorides combined in the same proportions as in Canquoin's caustic, form the only portion of M. Landolfi's preparation that is really active.

5. The chloride of bromine only acts by raising the epidermis, and exposing the denuded part to the action of the other two chlorides, a result easily obtained by any vesicatory applied just before employing Canquoin's paste.

6. M. Landolfi's preparation is, in fact, only this caustic masked by a coloring and odorous body, which, although it leaves the causticity unimpaired, destroys the precision of application. The chloride of bromine has only spoiled the mixture by rendering it fusible, much more difficult to manage, and much more uncertain in its results.

7. As the caustic so modified does not secure the patient from erysipelas or consecutive hæmorrhage, it can be no longer affirmed that its employment is exempt from danger.

8. Infinitely more painful than most others, this caustic induced most severe suffering, which, in general, lasts for six or eight hours, and may be prolonged for more than twenty-four hours. Opium and other narcotics are powerless against these pains, while their duration forbids our even thinking of employing anæsthetics.

9. The mode of application is quite vicious, and opposed to the rules of art. In place of attempting to at once destroy the cancerous tumor, M. Landolfi attacks it by partial and successive applications—a necessary consequence of employing a caustic the extent of the action of which cannot be calculated.

10. These successive applications, repeated on some patients fifteen or twenty times, induce a total amount of suffering hitherto unheard of.

11. They prolong the treatment indefinitely, and infinitely delay cicatrization.

12. The incessant irritation thus induced is of a nature to favor relapse, as experience has only too well shown, and all know who are imbued with sound surgical knowledge.

13. This method, applied by the inventor himself to nine cases of cancer of the breast and three cases of cancroid, has given the following results:—Of the 9 cases of cancer of the breast, 2 have died, 4 have suffered a notable aggravation of the disease, while in 3 cases in which cicatrization took place, the disease immediately after re-appeared; that is to say, in no case did a cure result. Of the 3 cases of cancroid, a cure took place in 1; in another there was cicatrization with re-appearance of the disease, and in the other, an exacerbation took place that necessitated the amputation of the limb.

To sum up, M. Landolfi's method can only be applied to certain cancers; it is more painful and more uncertain than several other modes of cauterization; and it is, in particular, inferior to Canquoin's method, of which it is only an altered copy. Like all the other methods of treatment, it may succeed in destroying certain tumors and cicatrization may follow; but it is quite powerless for the prevention of relapse, which it would seem rather to provoke, and so far from forming a step in advance, it adds but another to the illusions that so abound in the history of cancer.—*Med. Times and Gaz.*, from *Bull. de Therap.*

